# Role and mechanism of botanical drugs and their metabolites in osteoporosis: new strategies for clinical application

**DOI:** 10.3389/fphar.2025.1530194

**Published:** 2025-08-12

**Authors:** Xiujuan Yang, Yuqi Li, Jingjing Guo, Jiajia Wang, Shuo Li, Zhijun Yang, Pengxian Niu, Yiwei Jiang, Min Song, Yunxiang Hai

**Affiliations:** ^1^ Gansu University of Chinese Medicine, Lanzhou, China; ^2^ Northwest Collaborative Innovation Center for Traditional Chinese Medicine, Lanzhou, China; ^3^ Center of Traditional Chinese Medicine, Gansu, Lanzhou, China

**Keywords:** osteoporosis, bone, botanical drugs, metabolites, treatment

## Abstract

Osteoporosis, the most prevalent bone disease worldwide, is characterized by reduced bone mineral density and microarchitectural deterioration. Current pharmacological agents for osteoporosis management include bisphosphonates, calcitonin, estrogen, denosumab, and romosozumab. However, emerging evidence suggests these therapies may increase risks of breast cancer, ovarian cancer, osteonecrosis, and cardiovascular diseases. Consequently, safer therapeutic alternatives are required. Traditional botanical drugs, recognized for their favorable safety profiles compared to synthetic drugs, demonstrate increasing potential in osteoporosis treatment. This review examines classical pathogenic mechanisms of osteoporosis—including estrogen deficiency, oxidative stress, and dysregulated bone metabolism—and summarizes traditional botanical drugs: *Astragalus* polysaccharides (APS), glycyrrhizin, *Cistanche deserticola* polysaccharides (CDP), *Eucommia ulmoides* polysaccharides (EUP), and *Ligustrum lucidum* derivatives. These findings provide critical insights into osteoporosis pathomechanisms and identify promising therapeutic candidates for clinical translation.

## Graphical Abstract

**Figure:**
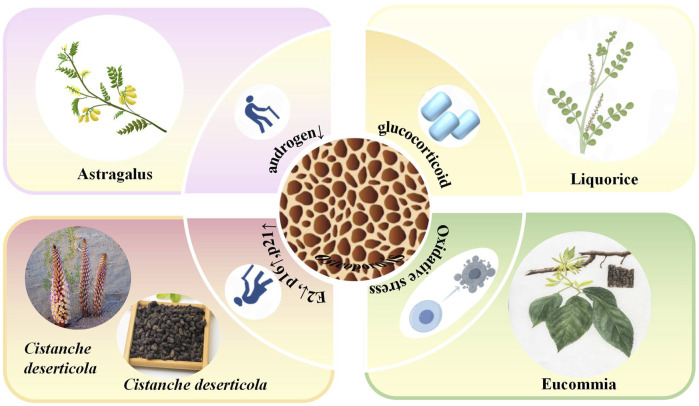
In this graphical abstract, we review the classical pathogenic mechanisms of osteoporosis, including estrogen deficiency, oxidative stress response, and bone metabolism. Based on these pathological mechanisms, this study summarizes the metabolites of several classic natural tonic plant drugs for the treatment of osteoporosis, including APS, glycyrrhizin, CDP, EUP, and the *Ligustrum lucidum* active metabolites. These findings may provide an important basis for clarifying the complex pathological mechanisms of OP.

## 1 Introduction

Osteoporosis (OP), a chronic skeletal disorder characterized by compromised bone strength, predisposes individuals to increased fracture risk (Rachner et al., 2011). According to WHO criteria, OP constitutes a systemic bone disease featuring reduced bone mass and microarchitectural deterioration, which elevates bone fragility and fracture susceptibility (Kanis, 1994). With accelerating global population aging, OP has evolved into a major public health challenge (Liu et al., 2018). A recent national epidemiological survey in China estimates approximately 90 million OP cases, with women comprising nearly 78% of this cohort (Marcella and Elizabeth, 2023). Consequently, OP substantially impairs patients’ quality of life while imposing considerable socioeconomic burdens (Nader et al., 2021). This review examines three principal pathogenic mechanisms underpinning OP: oxidative stress (Guadalupe et al., 2023), dysregulated bone metabolism (Schlaff, 2019), estrogen deficiency (Rani et al., 2023), and the roles of androgens, glucocorticoids, and aging.

Current osteoporosis pharmacotherapy primarily targets bone resorption inhibition, including bisphosphonates, calcitonin, and estrogen (Ian, 2021). However, long-term use of these agents may suppress bone remodeling and cause adverse effects such as medication-related osteonecrosis of the jaw (Ines et al., 2023). Denosumab, a monoclonal antibody against RANKL, specifically blocks RANKL-RANK interaction, thereby inhibiting osteoclast differentiation and reducing bone resorption (Chen et al., 2018; Kostenuik et al., 2011). Although exhibiting higher target specificity and extended half-life versus traditional therapies, denosumab carries risks of hypocalcemia and hypersensitivity (Trevino Manllo et al., 2014). Romosozumab, a sclerostin inhibitor, exerts dual effects by promoting bone formation via Wnt/β-catenin pathway activation (through LRP5/6 binding) while concomitantly decreasing resorption (Markham, 2019; Yang et al., 2020; Li et al., 2005). Despite superior fracture prevention efficacy versus bisphosphonates, romosozumab associates with increased cardiovascular event risk (Saag et al., 2017).

Traditional botanical drugs have been gradually accepted and recognized in the prevention and treatment of OP due to its low toxicity and side effects compared with synthetic drugs (Zhicai et al., 2022; Paschalis et al., 2017). Pharmacological studies have shown that metabolites produced by traditional botanical drugs can promote OBs, inhibit OCs, and regulate estrogen levels, as well as promote osteogenesis, inhibit adipogenesis in bone marrow mesenchymal stem cells (BMSCs), regulate calcium and phosphorus metabolism, and inhibit oxidative stress (OS) (Duan et al., 2023). Strong Bone Capsule is a classic commercial Chinese polyherbal preparation (CCPP) that has been extensively used in the clinical treatmentof primary OP in recent years. It has the functions of tonifying the kidney, strengthening tendons and bones, and promoting blood circulation (Wei et al., 2017). Its medicinal ingredient is the total flavonoids *Drynaria roosii* Nakaike [Polypodiaceae; *Drynaria roosii* Nakaike rhizoma]. It has no obvious toxic and side effects on the circulatory, nervous, and respiratory systems of animals. It can increase the levels ofsex hormones and gonadotropins and bone mineraldensity in rats, improve bonebiomechanical indexes, and has the effects of anti-inflammation, pain relief, and improvement of microcirculation. It also has apositive regulatory effect on the bone metabolism of OP rats (Huang A. Y. et al., 2022). In addition, studies have shown that Qianggu Capsule can significantly improvethe symptom of bone mass reduction in OP patients and has good safety (Guo and Wang, 2018). Clinical evidence has shown that it effectively improved BMD (Wei et al., 2017). Traditional botanical drugs have excellent pharmacological activities, a wide range of sources, few sideeffects, and a long history of use, and have shown good efficacy in various diseases (Qian et al., 2023). Meanwhile, various metabolites produced by botanical drugs have shown different biological effects in the treatment of osteoarthritic degenerative diseases, including OP. Many of these natural metabolites have demonstrated effects similar to those of traditional botanical drugs used in the treatment of OP (Monika et al., 2020). Many classic traditional botanical drugs have a long history of application. Reports about them can be found not only in numerous well-known medical classics but also in modern pharmacological studies. *Cistanche deserticola* Y. C. Ma [Orobanchaceae; *Cistanche deserticola* succulent stem] can tonify kidney-yang and is used to treat soreness and weakness of the waist and knees, as well as lack of strength in the muscles and bones (He et al., 1996). *Astragalus mongholicus* Bunge [Fabaceae; *Astragalus mongholicus* Bunge radix et rhizoma] has the efficacy of replenishing qi and strengthening the muscles and bones (Cao et al., 2012). *Ligustrum lucidum* Ait. [Oleaceae; *Ligustrum lucidum* fruit] can tonify the liver and kidney (Zhang et al., 2023). *Eucommia ulmoides* Oliv. [Eucommiaceae; *Eucommia ulmoides* bark] can treat low back pain and knee pain (Huang et al., 2021). *Glycyrrhiza glabra* L. [Fabaceae; *Glycyrrhiza glabra* L. radix et rhizoma] can strengthenthe muscles and bones and has estrogen-like effects (Cho and Kwun, 2018). In conclusion, the above five traditional botanical drugs can all be used to treat OP. In addition, through the summary of the literature, it can be seen that the metabolites of the above five traditional botanical drugs can also effectively promote the differentiation of OBs and inhibit the growth of OCs to varying degrees.

## 2 A systematic methodology

This review utilized multiple literature search strategies. Multiple authoritative databases were searched, including but not limited to PubMed (http://www.ncbi.nlm.nih.gov/pubmed), Web of Science (https://www.webofscience.com), Embase (https://www.embase.com/), China Knowledge (https://www.cnki.net/), Wanfang Data Knowledge Service Platform (http://www.wanfangdata.com.cn/), and Google Scholar (https://scholar.google.com). For “natural medicine metabolites,” “osteoporosis,” “bone density,” “bone metabolism,” “osteoblasts,” “osteoclasts,” “therapeutic effect” as keywords; at the same time, for the specific natural drug metabolite names “Glycyrrhizin,” “*Astragalus* polysaccharide,” “*Cistanche deserticola* polysaccharide,” “*Eucommia ulmoides* polysaccharide,” “*Ligustrum lucidum* active metabolites” was conducted. The searches were conducted using a combination of subject terms and free words, and were appropriately adjusted according to the characteristics of each database to ensure the comprehensiveness and accuracy of the searches. Literature that was not relevant to the study, had serious flaws in the experimental design, had incomplete data, or was not available in full text was excluded.

## 3 Pathogenetic mechanisms of OP

### 3.1 Bone immunology and metabolism

Bone homeostasis maintains a dynamic equilibrium between bone formation by OBs and resorption by OCs. In contrast, OP is characterized by a disrupted balance between bone formation and resorption (Huaqiang et al., 2021).

### 3.2 Osteoimmunology: bone-immune system interactions

Studies have increasingly reported a complex interplay between the skeletal and immune systems in the pathogenesis of osteoporosis (OP). This interdisciplinary relationship gave rise to the field of *osteoimmunology*, which emerged in the early 1970s, with the term formally introduced in Nature in 2000 (Arron and Choi, 2000; Huang F. et al., 2022). The concept of osteoimmunology highlights the bidirectional regulatory relationship between bone and immune systems, underscoring their mutual influence on physiological and pathological processes (Takayanagi, 2020). OBs and OCs activities are modulated by various soluble mediators secreted by immune cells, including cytokines, chemokines, and growth factors. Conversely, OBs and OCs also influence the behavior of hematopoietic stem cells, which give rise to diverse immune cell lineages (Giacomina et al., 2020). An expanding body of evidence demonstrates that both innate and adaptive immune cells contribute to OP development by releasing pro-inflammatory mediators (Janja et al., 2013; Mundy, 2007).

The discovery of the receptor activator of nuclear factor κB ligand (RANKL), its receptor RANK, and the decoy receptor osteoprotegerin (OPG) has firmly established the molecular foundation for the immune-bone axis. RANKL, primarily produced by osteoblasts, binds to RANK on OCs, initiating OCs differentiation and activation via the NF-κB, c-Jun N-terminal kinase (JNK), and protein kinase B (Akt) signaling pathways. OPG serves as a competitive inhibitor, preventing RANKL from engaging RANK and thus attenuating osteoclastogenesis (Marco and Nadia, 2019). Bone stromal cells are the principal source of membrane-bound RANKL, although a portion may be cleaved and released in soluble form (Chiara et al., 2014; Aseel et al., 2019). The expression of RANK and RANKL is notably prevalent in B cells and activated T lymphocytes. *In vitro* experiments have revealed that Janus kinase (JAK)1/2 inhibitors suppress OC formation by downregulating RANKL expression in osteoblasts (Katalin et al., 2019). A wide array of immune cells and cytokines critically modulate osteoblast development and bone remodeling through the RANKL/RANK/OPG axis, thereby reinforcing the integral role of immune mechanisms in skeletal homeostasis and the pathogenesis of OP (Tania et al., 2020).

Osteoimmunological research has demonstrated that pro-inflammatory cytokines play a pivotal role in promoting bone resorption through multiple mechanisms. These include inhibiting the differentiation of OBs, suppressing the activity of osteogenic proteins, upregulating RANKL expression by stromal cells, and directly enhancing osteoclast (OC)-mediated bone resorption. Among these cytokines, tumor necrosis factor (TNF) is recognized as the most critical inflammatory mediator contributing to osteolysis. Additionally, interleukin-6 (IL-6), interleukin-1 (IL-1), interleukin-17 (IL-17), and T helper 17 (Th17) cells have also been reported to exert similar pro-osteoclastic effects (Aseel et al., 2019).

These inflammatory factors primarily modulate key signaling pathways—most notably the RANK/RANKL/OPG axis and the Wnt signaling cascade—which are instrumental in regulating the function and activity of OBs and OCs. Activation or suppression of these pathways results in enhanced bone resorption and impaired bone formation, ultimately disrupting skeletal homeostasis and leading to progressive bone loss (Mina et al., 2020). Given that OP is fundamentally a disease mediated by immune system dysregulation, where immune activation drives the secretion of inflammatory mediators that inhibit OBs activity and promote OCs differentiation, future studies should prioritize investigating the immunological mechanisms underlying bone remodeling. Particular attention should be paid to the role of inflammatory cytokines in altering bone cell dynamics, as this may offer new insights into the pathogenesis and potential therapeutic targets of OP.

### 3.3 Mechanisms of OS driving OP

#### 3.3.1 OS foundation

OS characterized by the accumulation of oxidized substances in both intra- and extracellular environments, leads to an imbalance in redox homeostasis. It is recognized as a critical contributor to the pathophysiology of OP. Under normal physiological conditions, intracellular metabolic processes generate reactive oxygen species (ROS) as by-products, including superoxide anion (O_2_^−^), hydroxyl radical (HO^−^), and hydrogen peroxide (H_2_O_2_). To counteract the deleterious effects of ROS, the body employs an intricate antioxidant defense system composed of enzymatic and non-enzymatic components. Key antioxidant enzymes include superoxide dismutase (SOD), glutathione peroxidase (GPX), catalase (CAT), and the non-enzymatic antioxidant reduced glutathione (GSH), all of which act to neutralize ROS and maintain cellular redox equilibrium (Zhou et al., 2016).

#### 3.3.2 Effects of OS on OBs

##### 3.3.2.1 Bone reconstruction imbalance

The maintenance of skeletal homeostasis during bone remodeling depends on a dynamic equilibrium between bone formation and resorption, as well as the coordinated activity of bone-related cells, particularly osteoblasts (OBs) and osteoclasts (OCs). Aging disrupts this balance by impairing mitochondrial function and weakening the body’s antioxidant defense mechanisms, which collectively contribute to the accumulation of reactive oxygen species (ROS) (Jasreen et al., 2012). Elevated ROS levels increase mitochondrial membrane permeability, resulting in the release of cytochrome c—a key pro-apoptotic factor—into the cytosol, thereby initiating apoptosis. Concurrently, ROS levels in bone tissue rise with age and exert wide-ranging effects through the modulation of cytokines and signaling pathways. These pathways influence gene transcription and expression within the nucleus, promoting apoptosis in bone marrow mesenchymal stem cells (BMSCs) and OBs, while simultaneously enhancing the proliferation and differentiation of OCs. The net effect is a shift toward excessive bone resorption, culminating in the development of osteoporosis (OP) as a metabolic bone disorder characterized predominantly by bone loss (Robert et al., 2010). While low concentrations of ROS serve as physiological signaling molecules necessary for normal bone remodeling, excessive ROS disrupt this delicate balance and favor OCs-mediated bone degradation (Domazetovic et al., 2017).

##### 3.3.2.2 Enhanced bone resorption

*In vitro* experiments have demonstrated that ROS directly enhance the bone-resorbing activity of OCs (Cervellati et al., 2016). Furthermore, clinical studies have identified a positive correlation between serum OS marker levels and bone resorption rates in postmenopausal women (Gloria et al., 2018). These findings suggest that abnormally elevated ROS levels can significantly upregulate OCs activity, thereby contributing to increased bone degradation and the progression of OP.

##### 3.3.2.3 OS and OBs dysfunction

OS-induced apoptosis of OBs plays a pivotal role in the pathogenesis of glucocorticoid-induced and age-related OP (Takeshi et al., 2014). Among various reactive oxygen species (ROS), hydrogen peroxide (H_2_O_2_) has been shown to trigger OBs apoptosis by inducing mitochondrial dysfunction, particularly through disruptions in energy metabolism. This mitochondrial impairment further compromises bone formation capacity, thereby exacerbating skeletal fragility and contributing to osteoporotic progression (Panpan et al., 2017).

### 3.4 Hormone regulation and OP

#### 3.4.1 Estrogen deficiency

Estrogens—including estradiol, estriol, and estrone—regulate bone metabolism through both direct and indirect mechanisms mediated by estrogen receptors (ERs), primarily estrogen receptor alpha (ERα), which plays a predominant role in this process. Estrogen stimulates the transcription of Fas ligand (FasL) in OBs and promotes the generation of soluble FasL through cleavage of membrane-bound FasL by matrix metalloproteinase-3 (MMP3), ultimately inducing apoptosis of OCs (Aysha and Susan, 2016). Following menopause, estrogen levels decline sharply (Dechao et al., 2023), which is closely associated with diminished bone and muscle function, contributing to the development of both OP and sarcopenia (Zhe et al., 2021). A substantial body of evidence links estrogen deficiency to the pathogenesis of postmenopausal osteoporosis (PMOP) (Edward et al., 1996), and estrogen decline is considered a major factor driving OP in women after menopause. However, OP significantly compromises the quality of life in both men and women (The North American Menopause Society, 2021). Beyond its essential role in the female reproductive system, estrogen is also involved in the regulation of bone homeostasis, neural function, and inflammatory responses. Bone remodeling—a dynamic process involving OB-mediated bone formation and OC-mediated bone resorption—ensures the maintenance of skeletal integrity and metabolic balance (Tom et al., 2022). The activities of OBs and OCs are predominantly regulated by systemic hormones, particularly sex steroids such as estrogen (Ego, 2002).

Estrogen deficiency disrupts bone homeostasis by several mechanisms. It increases the activity of the basic multicellular unit (BMU), thereby shortening the bone formation phase, enhancing OBs apoptosis, and reducing OCs apoptosis—alterations that destabilize the OB-OC balance. Under physiological conditions, estrogen supports bone health by inhibiting OB apoptosis and promoting OCs apoptosis, thus maintaining a favorable ratio between bone formation and resorption (Schiavi et al., 2021).

Cell-based studies further support these observations. In MC3T3-E1 pre-osteoblastic cells, estrogen deficiency has been shown to impair cell differentiation and extracellular matrix synthesis (Di et al., 2021). Conversely, estrogen enhances osteoblast differentiation by activating the Wnt/β-catenin signaling pathway and upregulating bone morphogenetic protein 4 (BMP4) expression in OBs, thereby facilitating bone anabolism (Anna et al., 2020).

#### 3.4.2 Androgens and glucocorticoids

Sex hormone-binding globulin (SHBG) levels progressively increase with age and play a significant role in the pathogenesis of OP in men. Elevated SHBG reduces the levels of bioavailable testosterone and estradiol, thereby accelerating bone remodeling and enhancing bone resorption. Androgen receptors (ARs), expressed in OBs, OCs, and bone marrow stromal cells, also contribute directly to bone physiology. Androgens exert their bone-protective effects either directly—by stimulating OBs activity, including upregulation of alkaline phosphatase and osteocalcin expression—via AR signaling, or indirectly through aromatization into estrogen, which subsequently acts on estrogen receptors (Valdes et al., 2007; Christian et al., 1989).

**FIGURE 1:**
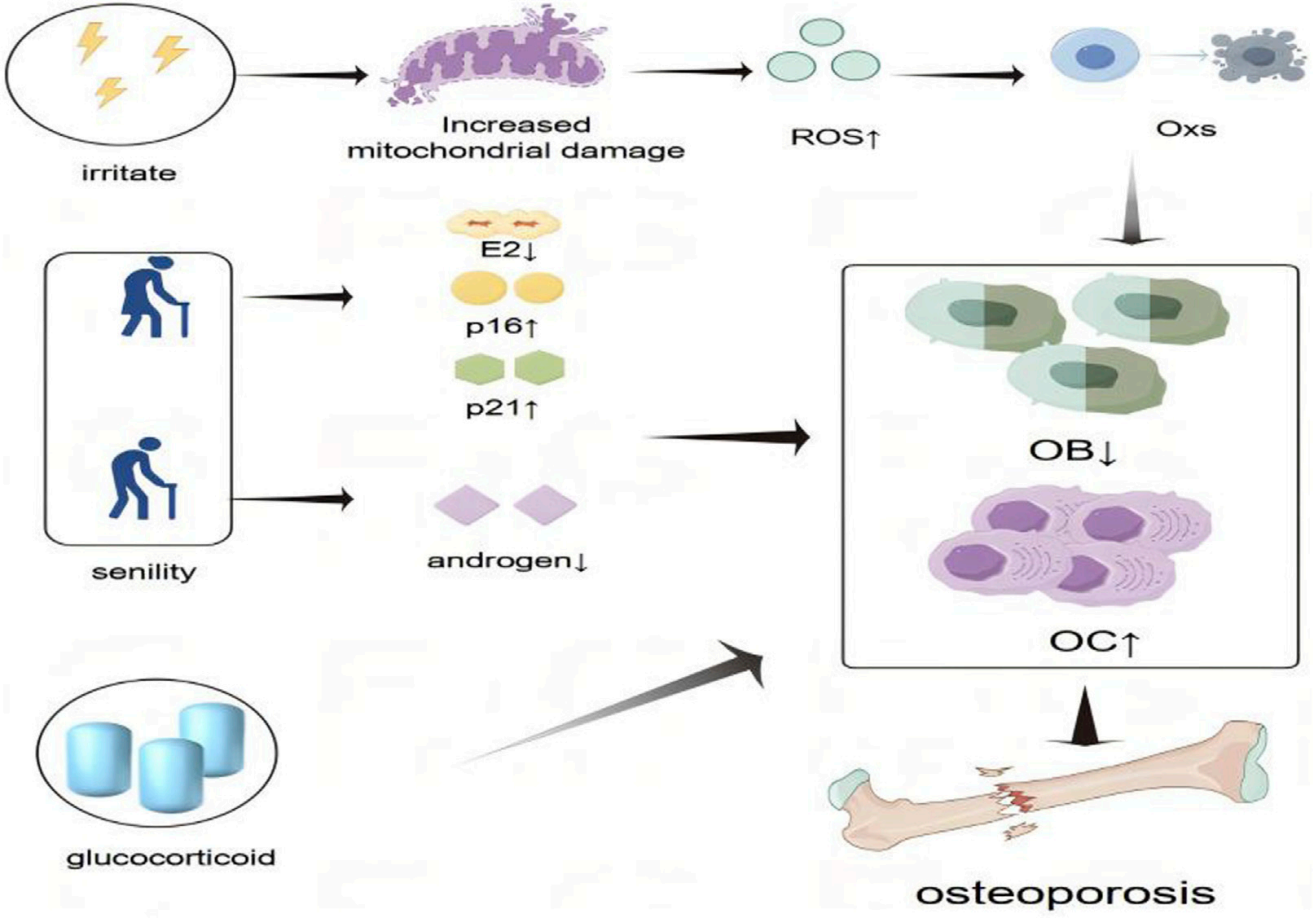
Schematic representation of the pathogenesis of osteoporosis. Osteoporosis is caused by an imbalance of bone homeostasis in the presence of various hormone deficiencies, immune imbalances, and oxidative stress. The “small upward arrow” indicates a rise or increase and the “small arrow down” indicates a decline or decrease.

Glucocorticoids (GCs) are extensively employed in the management of various inflammatory and autoimmune disorders. However, their long-term administration is associated with significant adverse effects, most notably glucocorticoid-induced osteoporosis (GIOP). GIOP, a form of secondary osteoporosis, is clinically characterized by progressive bone loss (Kennedy et al., 2006). Chronic GC exposure suppresses osteoblastogenesis, promotes apoptosis of osteoblasts and OBs, enhances OCs activity, and increases tissue sensitivity to parathyroid hormone, all of which contribute to the development of GIOP (Yamauchi, 2018; Canalis et al., 2007) (Figure 1). Given the multifactorial pathophysiology of OP—including dysregulated bone-immune crosstalk, oxidative stress, and hormonal alterations—natural metabolites have attracted increasing attention as potential therapeutic agents (The North American Menopause Society, 2021). Numerous natural compounds exhibit anti-osteoporotic properties by modulating immune-bone interactions, attenuating oxidative damage, influencing estrogen-mediated signaling pathways, and targeting other key mechanisms implicated in OP progression.

## 4 Botanical drugs metabolites in OP

Traditional botanical drugs have long been utilized for OP prevention treatment (Tang et al., 2023), such as *Morinda officinalis* How. [Rubiaceae; *Morinda officinalis* radix] mainly contains flavonoids and exerts preventive and therapeutic effects in OP by promoting bone differentiation, enhancing OCs viability, and promoting bone union to repair bone defects (Wu et al., 2024). In addition, *Cistanche deserticola* Y. C. Ma [Orobanchaceae; *Cistanche deserticola* succulent stem] can revitalize the liver and kidney, and strengthen muscles and bones (Shanshan et al., 2018). In recent years, in-depth research on traditional botanical drugs have provided evidence supporting the efficacy of traditional botanical drugs in the prevention and treatment of OP. From the perspective of Traditional Chinese Medicine (TCM), qi refers to the fundamental substance that constitutes the human body and sustains its life activities. Zhengqi represents the body’s innate immunity (Gao et al., 2023), and the onset of diseases is often attributed to a deficiency of Zhengqi. The term zangfu collectively refers to the five zang-organs and six fu-organs in the human body. Among these, the kidney, as one of the five zang-organs, plays a pivotal role in the development of OP (Zhang et al., 2011). Yin and Yang are concepts that generalize the opposing yet interconnected attributes of certain phenomena or elements in nature (Fu et al., 2021). When the balance between Yin and Yang is disrupted, the body becomessusceptible to disease. In summary, diseases arise when Zhengqi is insufficient, zangfu functions are impaired, and the balance between Yin and Yang is disturbed. In summary, diseases occur when the body’s Zhengqi is insufficient, the functions of the Zangfu are disordered, and the balance between Yin and Yang is disrupted. According to Traditional Chinese Medicine theory, the kidneys store essence (jing), which generatesbone marrow, nourishes the bones, strengthens them, and promotes their growth and repair (Wang et al., 2023). Kidney deficiency can lead to a decrease in estrogen levels, resulting in osteoporosis (Wang et al., 2016). Using botanical drugs can help alleviate this condition (Xie et al., 2023). Botanical drugs primarily function to strengthen Zhengqi, replenish deficiencies in qi, blood, Yin, and Yang, enhance physical constitution, boost immunity, alleviate symptoms of deficiency, and maintain normal physiological functions (Xie et al., 2023). From a TCM perspective, OP can be classified into syndromes such as liver and kidney Yin deficiency, or spleen and kidney Yang deficiency, among others (Xie et al., 2021). Studies have shown that natural metabolites from extracts of traditional botanical drugs such as *Astragalus mongholicus* Bunge [Fabaceae; Astragalus mongholicus Bunge radix et rhizoma], *Glycyrrhiza glabra* L. [Fabaceae; *Glycyrrhiza glabra* L. radix et rhizoma], *Cistanche deserticola* Y. C. Ma [Orobanchaceae; *Cistanche deserticola* succulent stem], *Eucommia ulmoides* Oliv. [Eucommiaceae; *Eucommia ulmoides* bark], and *Ligustrum lucidum* Ait. [Oleaceae; *Ligustrum lucidum* fruit] can be used to treat OP.

### 4.1 *Astragalus* polysaccharide (APS)

*Astragalus mongholicus* Bunge [Fabaceae; *Astragalus mongholicus* Bunge radix et rhizoma] comprises over 2,000 species distributed worldwide (Roohi et al., 2020). Studies have demonstrated that *Astragalus mongholicus* Bunge [Fabaceae; *Astragalus mongholicus* Bunge radix et rhizoma] can exert estrogen-like effects and significantly improved the serum levels of inflammatory factors such as interleukin-2 (IL-2) and interleukin-8 (IL-8) in ovariectomized female rats. However, no significant change was observed in the serum levels of estradiol, follicle-stimulating hormone, luteinizing hormone, and other sex hormones (Yan et al., 2012). *Astragalus* polysaccharide (APS) is an important bioactive metabolite and is derived from the dried roots of *Astragalus mongholicus* Bunge [Fabaceae; Astragalus mongholicus Bunge radix et rhizoma] (Li et al., 2022). APS is a major active ingredient of *Astragalus mongholicus* Bunge [Fabaceae; *Astragalus mongholicus* Bunge radix et rhizoma] (Li et al., 2022), which can alleviate the symptoms of PMOP (Kong et al., 2012; Li et al., 2019). APS has estrogen-like effects, increasing bone mass, decreasing serum ALP and BGP values, increasing blood calcium levels, and increasing femur and vertebrae bone density in rats. Meanwhile, a study on ovariectomized rats revealed that APS also inhibits the gene expression of *FoxO3a* mRNA by increasing the gene expression of β-catenin and Wnt2 mRNA, thereby increasing the bone mineral content of the femur, increasing the maximum stress, maximum load, and elastic modulus, and improving the OS condition. APS can restore intestinal function by reversing gene expression in osteoporotic rats. Moreover, APS determines the reprogramming of intestinal function to attenuate OP through the intestinal osteoblastic axis (Junsheng et al., 2021).

### 4.2 Glycyrrhizin

*Glycyrrhiza glabra* L. [Fabaceae; *Glycyrrhiza glabra* L. radix et rhizoma] is a common traditional botanical drug (Shang et al., 2022). Glycyrrhizin is the most potent triterpenoid saponin glycoside constituent of *Glycyrrhiza glabra* L. [Fabaceae; *Glycyrrhiza glabra* L. radix et rhizoma], which possesses anti-inflammatory, anti-tumor, anti-aging, and antioxidant properties (Ekanayaka et al., 2018; Vikram et al., 2017). Glycyrrhizin effectively inhibited RANKL-induced OCs ogenesis *in vitro*. Meanwhile, glycyrrhizin was found to reduce bone resorption in a dose-dependent manner, resulting in effective suppression of OP. RT-PCR and Western blotting revealed that the expression of osteoclast-related genes, including NFATc1, c-FOS, TRAP, CK, CTR, DC-STAMP, and OSCAR, was significantly reduced in a dose-dependent manner (Nada et al., 2018). Therefore, Glycyrrhizin attenuates RANKL-induced oxidative stress in OCs, thereby inhibiting osteoclastogenesis by activating the AMPK/Nrf 2 axis and reducing ROS in OCs. Lowering the ROS levels represents a therapeutic approach, and glycyrrhizin can be used as a potent antioxidant agent for the treatment of OP and bone resorption (Xuan et al., 2017).

### 4.3 *Cistanche deserticola* polysaccharide (CDP)

*Cistanche deserticola* Y. C. Ma [Orobanchaceae; *Cistanche deserticola* succulent stem] is a parasitic plant mainly found in the desert areas of the northwestern part of China (Zhiming et al., 2016). It is a widely used tonic medicinal food in China and has been proven to be effective in treating OP (Yang et al., 2024), and rich in active ingredients such as pine mullein and fructuschrysoside, which exert a variety of pharmacological activities such as bone protection, anti-aging, and antioxidant (Wang et al., 2012). Among them, CDP is a polysaccharide extracted from *Cistanche deserticola* Y. C. Ma [Orobanchaceae; *Cistanche deserticola* succulent stem] (Xiao et al., 2021), CDP suppresses RANKL-induced OCs differentiation. A potential mechanism for the treatment of OP with CDP is the enhanced expression of antioxidant enzymes, thereby reducing the production of ROS and inhibiting RANKL-activated NFAT and mitogen-activated protein kinase (MAPK) signaling cascade responses (Dezhi et al., 2018). Based on the above findings, CDP can be used to treat OP (Zhang et al., 2019). In addition, the metabolites of *Cistanche deserticola* Y. C. Ma [Orobanchaceae; *Cistanche deserticola* succulent stem] have been shown to increase the levels of osteocalcin, calcium ions, and serum ALP and promote the expression of bone morphogenetic protein 2 (BMP2) in OBs in rats (Fujiang et al., 2021). The total glycosides and polysaccharide active ingredients in the total *Cistanche deserticola* Y. C. Ma [Orobanchaceae; *Cistanche deserticola* succulent stem] metabolites significantly decreased the expression of NF-κB receptor activator ligand (RANKL) and p-β-catenin, while up-regulating the expression of OCN, BMP2, OPG and P-GSK-3β (Ser 9). Furthermore, the total metabolites of *Cistanche deserticola* Y. C. Ma [Orobanchaceae; *Cistanche deserticola* succulent stem] also promoted bone formation in OBs and improved the healing of bone microstructural damage in SAMP6 mice (Cheng et al., 2022). CDP prevented the ameliorative loss induced by excision of warm nests by inhibiting OCs activity function.

### 4.4 *Eucommia ulmoides* polysaccharide (EUP)

*Eucommia ulmoides* Oliv. [Eucommiaceae; *Eucommia ulmoides* bark] is a classic traditional botanical drug used in the prevention and treatment of OP (Pan et al., 2014). The active metabolites of *Eucommia ulmoides* Oliv. [Eucommiaceae; *Eucommia ulmoides* bark] include flavonoids, lignans, and myricetin, which have been proven to exert beneficial effects against OP (Xirui et al., 2014). Among them, EUP is an acidic polysaccharide isolated and purified from *Eucommia ulmoides* Oliv. [Eucommiaceae; *Eucommia ulmoides* bark] (Jiyu et al., 2023). EUP has been shown to significantly ameliorate microstructural damage in the bone tissue of osteoporotic mice. Specifically, EUP treatment increased both the number and surface area of OBs within bone tissue and enhanced the expression of OBs differentiation-related proteins. Concurrently, it reduced the number and surface area of OCs and downregulated the expression of osteoclast-associated proteins (Huang et al., 2021). These findings suggest that EUP effectively restores the balance of bone remodeling in osteoporotic conditions. To further elucidate the underlying mechanisms of EUP in modulating bone metabolism, serum enzyme-linked immunosorbent assay and Western blot analysis of bone tissue were conducted. The results demonstrated that EUP alleviates OS via activation of the ERK/JNK/NRF2 signaling cascade and promotes osteogenesis through the bone morphogenetic protein-2 (BMP-2)/Smad signaling pathway. These pathways collectively contribute to the enhancement of bone metabolic activity in osteoporotic mice (Jiyu et al., 2023). Collectively, the above findings indicate that EUP exerts therapeutic effects on OP in murine models by restoring cortical bone thickness, increasing mineralized bone area, elevating OBs numbers, and reducing OCs presence along the cortical bone surface.

### 4.5 *Ligustrum lucidum* active metabolites

**FIGURE 2:**
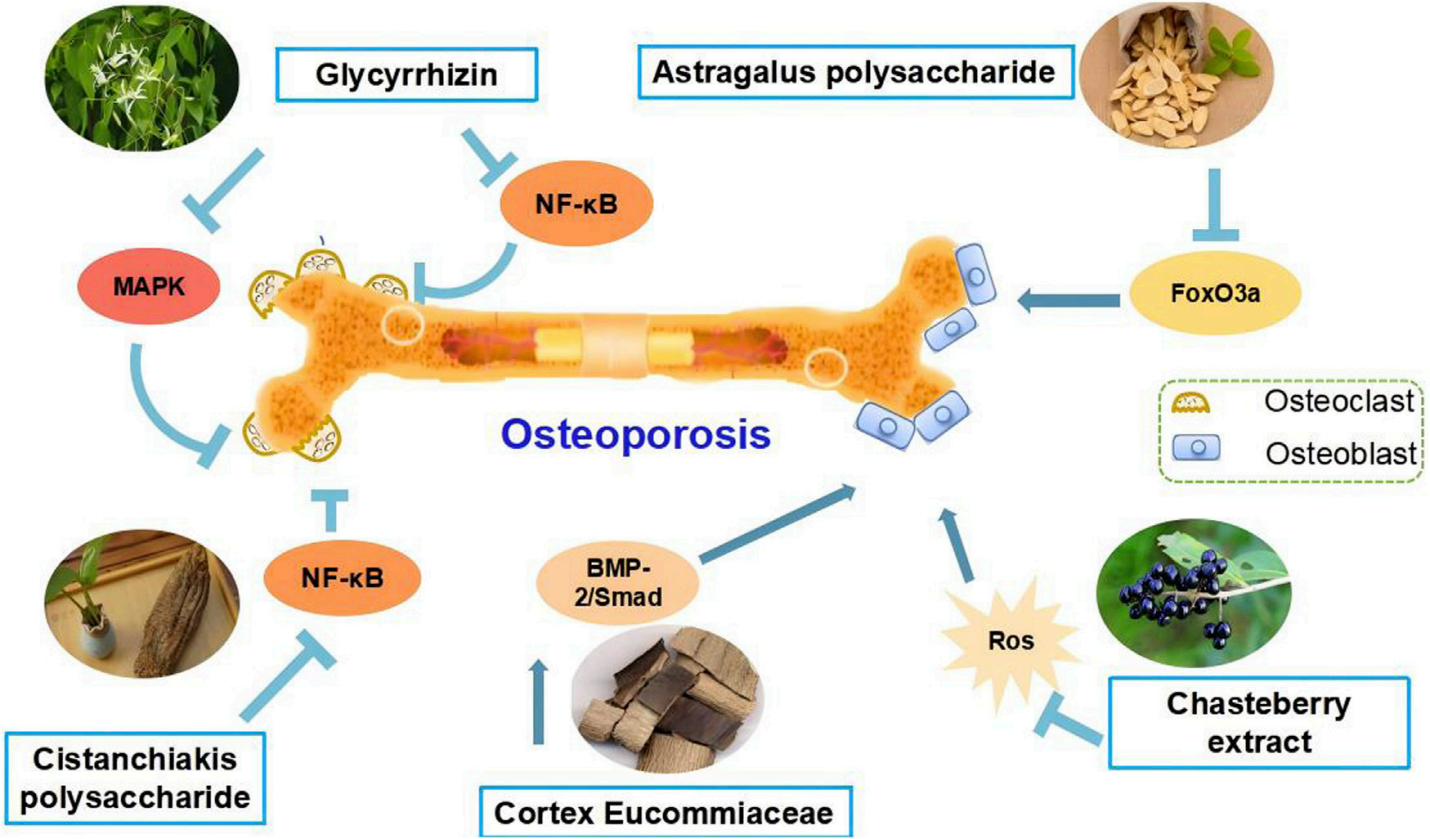
Schematic representation of the mechanism of anti-osteoporotic effects of natural plant compounds on osteoblasts and osteoclasts. Activation of MAPK, Wnt/β-catenin signalling pathways or inhibition of NF-κB, RANKL/RANK signalling pathways can promote the proliferation or differentiation of osteoblasts, which is beneficial for the treatment of osteoporosis. Arrows indicate activation of factors or positive effects on the indicated cell types, while inverted T marks indicate inhibition or negative effects.

*Ligustrum lucidum* Ait. [Oleaceae; *Ligustrum lucidum* fruit] is a traditional botanical drug that has been used for tonifying kidney and liver decades (Zhang et al., 2023). Steaming *Ligustrum lucidum* Ait. [Oleaceae; *Ligustrum lucidum* fruit] can moderate the coolness and slipperiness of *Ligustrum lucidum* Ait. [Oleaceae; *Ligustrum lucidum* fruit], and enhance the tonic on the liver and kidney (Ruqiao et al., 2021). It is a commonly used botanical drugs remedy for age-related conditions such as OP (Xiaoyan et al., 2023). The National Health Commission of the People’s Republic of China clearly categorized *Ligustrum lucidum* Ait. [Oleaceae; *Ligustrum lucidum* fruit] as a traditional botanical drug that can be used in nutritional supplements (Zhang et al., 2006). Some studies on de-ovulated rats reported that *Ligustrum lucidum* Ait. [Oleaceae; *Ligustrum lucidum* fruit] metabolites can regulate bone turnover (Yan et al., 2008a), improve calcium homeostasis (Yan et al., 2008b), and bone properties and prevent bone loss (Yi et al., 2021). Early intervention to attenuate bone loss in postmenopausal women can effectively slow down the development of OP, which is of great significance for the prevention and treatment of PMOP. *Ligustrum lucidum* Ait. [Oleaceae; *Ligustrum lucidum* fruit] metabolites were also found to inhibit adipogenesis in ovariectomized mice. It is a potential candidate for the prevention and treatment of PMOP, which nourishes the liver and kidneys (Black and Rosen, 2016). Research on ovariectomized rats revealed that it exerts beneficial effects on bone turnover and calcium homeostasis. Moreover, *Ligustrum lucidum* Ait. [Oleaceae; *Ligustrum lucidum* fruit] showed significant preventive effects against OP by inhibiting Oxs, increasing BMD, improving bone microstructure, and promoting osteoblast proliferation and OPG protein expression, but had no therapeutic effect on bone loss in aged mice (Xiaoyan et al., 2023). Rhodioloside is one of the main metabolites of *Ligustrum lucidum* Ait. [Oleaceae; *Ligustrum lucidum* fruit] aqueous extract and can activate the Wnt/β-catenin signaling pathway, thereby promoting the differentiation of mouse BMSCs into neuronal cells (Ko et al., 2010). The active ingredients of *Ligustrum lucidum* Ait. [Oleaceae; *Ligustrum lucidum* fruit] can increase mineral density and produce preventive therapeutic effects by promoting osteogenic cell differentiation, inhibiting the expression of matrix metalloproteinase, and hindering OCs activation (Chen et al., 2017) (Figure 2).

## 5 Conclusion and perspectives

Osteoporosis (OP), characterized by compromised bone strength and elevated fracture risk, constitutes a global health burden exacerbated by population aging, requiring long-term management that increases mortality and healthcare costs. Traditional botanical drugs exemplified by *Astragalus mongholicus* Bunge [Fabaceae; *Astragalus mongholicus* Bunge radix et rhizoma], *Glycyrrhiza glabra* L. [Fabaceae; *Glycyrrhiza glabra* L. radix et rhizoma], *Cistanche deserticola* Y. C. Ma [Orobanchaceae; *Cistanche deserticola* succulent stem], *Eucommia ulmoides* Oliv. [Eucommiaceae; *Eucommia ulmoides* bark], and *Ligustrum lucidum* Ait. [Oleaceae; *Ligustrum lucidum* fruit] demonstrating therapeutic potential against core OP pathomechanisms including oxidative stress, dysregulated bone metabolism, and estrogen deficiency. Specifically, APS enhances femoral mineral density via Wnt/β-catenin activation. Glycyrrhizin suppresses osteoclastogenesis through AMPK/NRF2-mediated ROS reduction; CDP inhibits RANKL-induced OCs differentiation via NFAT/MAPK pathway blockade; EUP promotes osteoblast ogenesis through BMP2/Smad signaling while ameliorating oxidative stress via ERK/JNK/NRF2; and *Ligustrum lucidum* active metabolites activate Wnt/β-catenin to drive BMSC osteogenic differentiation. These metabolites restore bone homeostasis by modulating OBs/OCs dynamics, yet face pharmacokinetic limitations including narrow metabolite coverage, low bioavailability, and non-targeted tissue accumulation that may induce hepatorenal toxicity. Nanomaterial-based delivery systems (e.g., polymeric nanoparticles) offer solutions by enhancing stability and enabling controlled release to minimize off-target effects (Hubbell and Chilkoti, 2012), while precision dosing strategies must mitigate clinical adverse events (e.g., *Epimedium* total flavonoids) (Li et al., 2024). Robust randomized controlled trials remain imperative to validate efficacy and safety profiles.

To bridge these gaps, future research should be deepened in the following aspects: (1) establishing validated osteoporosis animal models that integrate TCM syndrome differentiation with modern phenotyping techniques; (2) systematically elucidating the pharmacokinetic profiles and multi-organ regulatory mechanisms (e.g., gut-bone axis) of natural metabolites; (3) implementing comprehensive safety assessments per OECD/ICH guidelines, particularly chronic hepatorenal toxicity and genotoxicity risks; and (4) translating lead natural metabolites demonstrating>50% oral bioavailability into multi-center, dose-optimized randomized controlled trials for efficacy validation.

## Glossary

ACP5: Anti-APC5
ALP: alkaline phosphatase
APS: aminopeptidases
APS: *Astragalus* polysaccharide
BGP: bone-γ-carboxyglutamic acid-containing protein
BMD: bone mineral density
BMP2: bone morphogenetic protein 2
BMP-4: bone morphogenetic protein
BMU: basic multicelluler unit
CDP: *Cistanche deserticola* polysaccharide
c-FOS: cellular oncogene fos
CK: cytokeratin
CTR: calcitonin receptor
CTSK: recombinant cathepsin K
DC-stamp: dendritic cell-specific transmembrane protein
ERK: extracellular regulated protein kinases
EUP: *Eucommia ulmoides* polysaccharide
FasL: fas ligand
GCLC: recombinant glutamate cysteine ligase catalytic
GIOP: glucocorticoid-induced osteoporosis
**H**_**2**_ **O**_**2**_: hydrogen peroxide
HO-1: heme oxygenase 1
IL-2: interleukin-2
IL-8: interleukin-8
JNK: c-JunN-terminalkinase
MAPKs: mitogen-activated protein kinases
M-CSF: macrophage colony-stimulating factor
MMP3: matrix metalloproteinase 3
MSCs: bone marrow mesenchymal stem cells
NFATc 1: nuclear factor of activated T cells 1
NFATC1: recombinant nuclear factor of activated T-cells, cytoplasmic 1
NQO1: NAD(P)H: quinoneoxidoreductase
NQO1: recombinant human NQO1 protein
NRF2: nuclear factor erythroid 2-related factor 2
OB: osteoblasts
OCs: osteoclasts
OP: osteoporosis
OPG: osteoprotegerin
Os: oxidative stress
OSCAR: dendritic cell-specific transmembrane protein
PMOP: postmenopausal osteoporosis
POP: primary osteoporosis
ROS: reactive oxygen species
TCM: traditional Chinese medicine
TRAP: translocon-associated protein
WHO: World Health Organization
